# Elephant Management in North American Zoos: Environmental Enrichment, Feeding, Exercise, and Training

**DOI:** 10.1371/journal.pone.0152490

**Published:** 2016-07-14

**Authors:** Brian J. Greco, Cheryl L. Meehan, Lance J. Miller, David J. Shepherdson, Kari A. Morfeld, Jeff Andrews, Anne M. Baker, Kathy Carlstead, Joy A. Mench

**Affiliations:** 1 Department of Animal Science and Center for Animal Welfare, University of California Davis, Davis, California, United States of America; 2 AWARE Institute, Portland, Oregon, United States of America; 3 Chicago Zoological Society—Brookfield Zoo, Brookfield, Illinois, United States of America; 4 Oregon Zoo, Portland, Oregon, United States of America; 5 Smithsonian Conservation Biology Institute, National Zoological Park, Front Royal, Virginia, United States of America; 6 CBSG North America, Apple Valley, Maine, United States of America; 7 Busch Gardens, Tampa, Florida, United States of America; 8 Honolulu Zoo, Honolulu, Hawaii, United States of America; University of Tasmania, AUSTRALIA

## Abstract

The management of African (*Loxodonta africana*) and Asian (*Elephas* maximus) elephants in zoos involves a range of practices including feeding, exercise, training, and environmental enrichment. These practices are necessary to meet the elephants’ nutritional, healthcare, and husbandry needs. However, these practices are not standardized, resulting in likely variation among zoos as well as differences in the way they are applied to individual elephants within a zoo. To characterize elephant management in North America, we collected survey data from zoos accredited by the Association of Zoos and Aquariums, developed 26 variables, generated population level descriptive statistics, and analyzed them to identify differences attributable to sex and species. Sixty-seven zoos submitted surveys describing the management of 224 elephants and the training experiences of 227 elephants. Asian elephants spent more time managed (defined as interacting directly with staff) than Africans (mean time managed: Asians = 56.9%; Africans = 48.6%; *p*<0.001), and managed time increased by 20.2% for every year of age for both species. Enrichment, feeding, and exercise programs were evaluated using diversity indices, with mean scores across zoos in the midrange for these measures. There were an average of 7.2 feedings every 24-hour period, with only 1.2 occurring during the nighttime. Feeding schedules were predictable at 47.5% of zoos. We also calculated the relative use of rewarding and aversive techniques employed during training interactions. The population median was seven on a scale from one (representing only aversive stimuli) to nine (representing only rewarding stimuli). The results of our study provide essential information for understanding management variation that could be relevant to welfare. Furthermore, the variables we created have been used in subsequent elephant welfare analyses.

## Introduction

There is significant interest within and beyond the zoological community in understanding the management of Asian (*Elephas maximas*) and African (*Loxodonta africana*) elephants in zoos, particularly as it relates to evaluating practices that are relevant to welfare [1,2], such as feeding, training, exercise, husbandry and environmental enrichment. While these practices are required elements of all elephant programs accredited by the Association of Zoos and Aquariums (AZA) [3], they are not standardized. Because they are not standardized, there may be significant variation among zoos with respect to daily care, which could in turn affect elephant welfare.

Assessing elephant management at the population level requires the development of systematic methods for measuring and quantifying these varying practices. However, little research has been conducted that either describes management practices or compares them across multiple zoos. This lack of research on management has been cited as one of the major impediments to formally assessing the welfare of zoo animals [4].

In addition to gaining an understanding of the management of zoo elephants in general, it is also important to understand how management practices differ between African and Asian elephant zoo populations. One might expect the two species to be managed differently given that they are adapted to different ecological niches [5,6] and may have different life history characteristics. For example, a larger proportion of Asian elephants (38%) than African elephants (21%) in North American zoos are captive born [7] and, for those elephants imported from home range countries, Asian elephants are more likely to have previous captive experiences than African elephants [8]). Similarly, one might also expect management practices to be shaped by the different physiological (*e*.*g*., size, reproductive cycles, metabolic demands) and behavioral (*e*.*g*., social development and group dynamics) characteristics of female and male elephants [5].

This paper focuses on the development and analysis of management variables relating to feeding, exercise, training and enrichment. The dual purpose was to provide a comprehensive review of elephant management in zoos and to generate appropriate independent variables to be used in subsequent epidemiological analyses of behavioral [9–11], physiological [12], and health-related [13,14] welfare indicators. A similar approach was taken in a related paper that characterizes the housing and social management of these same elephants [15].

Our study focused on feeding, enrichment, exercise and training, because research in many species demonstrates that animals’ experiences of these components of managed care play a critical role in their welfare. In the remainder of the introduction, we present the rationale for investigating each management category in the context of welfare assessment.

### Management Schedules

Management routines can vary considerably at the zoo and individual elephant level depending on staffing, management philosophy, and the needs of each elephant. However, AZA accreditation standards [3] require that all elephants participate in an exercise program, behavior training, and a variety of husbandry activities including baths, foot and skin care, and physical assessments. Each of these activities requires staff to interact directly with the elephants. When not interacting with staff members, elephants spend time independently, housed either alone or in social groups of varying sizes and compositions [15] and in enclosures that are visible (on exhibit) or not visible (off exhibit) to the public.

Management schedules may have important implications for elephant welfare. For example, elephants who spend more time interacting with staff may perceive healthcare or medical procedures as less stressful, as demonstrated by studies conducted with other species (*e*.*g*., chimpanzees [16], cats [17]). Conversely, elephants who spend more time independently may have more opportunity to exercise choice and behave autonomously, both of which have been suggested to enhance welfare [18,19]. Thus, we were interested in characterizing the percentage of daytime hours elephants spend interacting directly with staff and the amount of time spent during those hours in either training, exercise, husbandry, play, or public demonstrations (in an education or show setting).

### Environmental Enrichment

Within the zoo and aquarium community, the term enrichment (or environmental enrichment) covers a wide range of practices intended to improve animal welfare by facilitating the expression of important behaviors, such as foraging or self-maintenance, and by providing opportunities for play, exploration, problem solving, and exercising choice [20,21]. Enrichment in zoos can take many forms, ranging from permanent or semi-permanent exhibit features, such as the provision of pools, items that require animals to use an array of skills to access food (*e*.*g*., food-balls, puzzles), or other rewarding stimuli [21]. Numerous studies with various species of zoo animals have demonstrated the positive impact of environmental enrichment on welfare outcomes including reductions in stereotypic behavior [22,23] and physiological stress responses [24]. However, there are very few published studies of enrichment practices for elephants, and those focus exclusively on food-based enrichment at single zoos (*e*.*g*., Stoinski et al. [25],Wiedenmayer [26]).

Our goal was to determine the enrichment methods used by zoos. We also wanted to assess whether enrichment was applied systematically, which can enhance its effectiveness [27]. This inquiry was informed by the work of Mellen and Sevenich MacPhee [27], who proposed the “SPIDER” framework. This model, which starts with Setting Goals and proceeds through Planning, Implementation, Documenting, Evaluation, and Readjustment, presents a framework that emphasizes the importance of regularly assessing enrichment for its biological relevance and its ability to engage the animals.

### Feeding

As large herbivores, elephants spend a significant proportion of time (in the wild [5,6] and in zoos [28]) manipulating and consuming plant material to meet metabolic needs. In the wild, food resources are temporally and spatially dispersed, and elephants use a range of foraging skills to locate and access them [5,6]. Given the fact that the acquisition and ingestion of food plays such a central role in elephant behavioral repertoire, the timing, frequency, and methods of food provision are important components of zoo feeding programs. In zoos, feeding opportunities are, of necessity, spatially and temporally concentrated [25], but it has been suggested that this limitation can be addressed by utilizing a diversity of feeding methods and schedules [29,30]. To better understand the non-nutritional aspects of elephant feeding programs, we sought to determine the patterns in and diversity of food delivery methods. In addition, we were interested in the temporal predictability of food presentation. Although the effects of feeding predictability on elephant welfare have not been evaluated, temporally unpredictable feeding schedules have been shown to improve welfare in other species by increasing exploratory behavior, enhancing memory, and reducing the performance of stereotypic behavior [31,32].

### Exercise

AZA accreditation standards require that all zoos have a staff-directed elephant exercise program [3]. Elephants can be trained to participate in exercise routines including activities such as stretching and staff-directed walking. Exercise programs have been implemented, in part, because zoo elephants are prone to developing *pododermatitis* and degenerative bone disease [33,34]. Many zoo elephants are also overweight or obese [14]. Research in humans [35–38] and other species (rodents [39–42], dogs [43–45], swine [46]) demonstrates that exercise can reduce the risk of developing health issues and mitigate complications associated with these ailments once developed. Our goal was to assess the frequency and types of staff-directed exercise employed.

### Training

Care staff train elephants to participate in a number of management practices, including daily foot and skin care, moving between exhibit areas on cue, and veterinary procedures. Elephant training involves teaching an individual elephant to perform, or abstain from performing, behaviors on request [47]. However, the methods by which elephants are trained in zoos across North America vary considerably depending on the philosophy and experience of the trainers as well as the age, species, sex, and background of the individual elephant (for a review, see Wemmer and Christen [8]). Although the specific techniques and tools used may vary, all elephant training is based on the application of operant conditioning methods that are designed to either: 1) increase the frequency of desired behaviors, typically by either presenting stimuli the animal finds rewarding or removing stimuli the animal finds aversive or 2) decrease the frequency of undesired behaviors, typically by presenting aversive stimuli or removing rewarding stimuli [48,49]. Additionally, trainers may use neutral techniques (*e*.*g*., taking a short pause or not reacting to the elephant’s behavior) when they wish to provide the animal with another opportunity to be successful [50].

The effects of utilizing rewarding and aversive stimuli in the learning environment have been investigated with humans and other species. For example, humans who learn tasks under conditions where aversive stimuli are utilized are more likely to experience negative affective states and/or frustration than those who are taught tasks with only the use of rewarding or neutral stimuli [51,52]. Similarly animals trained with aversive stimuli show behavioral and neurological responses considered indicative of negative affect [51,53,54].

There has been no systematic investigation into how the techniques and tools utilized in elephant training influence welfare. However, controversy has arisen with regard to one of the tools used for training elephants: the pole/stick, called a guide. The guide is also known as an ankus or bullhook, since there is a metal hook with a pointed tip at the distal end. Some speculate that guide use creates an aversive training environment, because of its potential to cause physical discomfort [55,56], while others contend that its use has few adverse welfare consequences if rewarding stimuli are used more frequently than the guide during training [47]. Since the methods utilized by elephant trainers have never been rigorously documented these assertions cannot be tested empirically. Thus, our goals were to collect data on the use of guides and operant conditioning techniques and to develop variables to characterize the different approaches to elephant training across the North American zoo population.

## Methods

### Ethics statement

The management at each participating zoo authorized this study, and the University of California, Davis Institutional Review Board determined that the surveys used in this study did not constitute Human Subject Research (IRB #739963–1).

### Surveys

We derived the data from two online surveys, referred to as the Management Survey and the Training Techniques Survey. Survey content was developed from multiple sources including expert opinion, focus group interviews with elephant care professionals, and current elephant management literature. For both surveys, we asked respondents to report on their practices during the 2012 calendar year. Online surveys were generated dynamically based on response-dependent branching architecture to avoid presenting redundant or inapplicable questions to respondents. All data were stored upon submission in a relational database using an SQL server, and we ensured confidentiality by using randomly generated unique alpha-numeric codes.

We used customized web-links to invite elephant managers to participate in the Management Survey. The survey included five sections, each consisting of one to five questions. Two of these sections collected information at the zoo level (enrichment and feeding), while three collected information at the individual elephant level (management schedules, use of training tools, and exercise). Questions at the individual elephant level were presented in the survey sequentially for each elephant at the zoo and were based on elephant population data that we collected prior to developing the survey.

We targeted the Training Techniques Survey at elephant care staff, who had worked with a given elephant for at least one year. Elephants were randomly matched with between one and four qualified care staff respondents depending on the elephant to care staff ratio at that zoo. The survey characterized each individual elephant’s training experience by focusing on techniques used during training both when the elephant was compliant and non-compliant. Response choice ranged from Never (1) to Very Frequently (5) on a 5-point Likert scale. A complete list of the Training Techniques Survey questions can be found in Tables 1 and 2.

**Table 1:** Survey Question: Please indicate the frequency with which you utilize each of the following training techniques when the elephant complies with a training request.

Training technique	Definition
Ask	Ask for another behavior
Food	Give food that the elephant likes
No Response	Do nothing
Pat	Pat, rub, or scratch the elephant
Remove Negative	Remove item(s) the elephant does not like, *e*.*g*., contact with a guide, shift dominant animals away
Toy	Give other item(s) the elephant likes (ice, toy, etc.)
Verbal	Give verbal praise or use a clicker or whistle

**Table 2:** Survey Question: Please indicate the frequency with which you utilize each of the following training technique when the elephant does not comply with a training request.

Training technique	Definition
Different	Request a different behavior
Forceful Pressure	Apply forceful pressure with the guide or other object
Gentle Pressure	Apply gentle pressure with the guide or other object
No	Say "No"
No Food	Do not give food reinforcement
Pause	Give a neutral pause for 3–5 seconds
Remove Likes	Remove items that the elephant likes (toy, social partner, etc.)
Repeat	Request the behavior again
Show Negative	Show the guide without making contact
Swat	Apply a swat with the guide or other object
Target	Request the same or other behavior, aiding the animal by encouraging approach towards a target (*e*.*g*., a pole, brush, hand, or other item).
Time Out	Remove attention and opportunities for reward

### Data Processing and Variable Creation

We excluded all data from partially completed sections of the surveys. Data from elephants that were born, died, or experienced an inter-zoo transfer during the study year were also excluded from all analyses. We used the data to create a number of novel variables characterizing management schedules, environmental enrichment, feeding, exercise, and training practices. Some of these variables describe raw data and others were calculated to synthesize composite values. Throughout the remainder of this paper, we capitalize all calculated and synthesized variables. Variable descriptions are presented below and in Tables 3 and 4.

**Table 3:** Variables created from the Management Survey.

Variable name	Unit of analysis	Description	Data type and possible range (min, max)
Percent Time Off Exhibit	Elephant	Percent time spent in spaces that are not viewable by the public	Percentage: Range (0, 100)
Percent Time On Exhibit	Elephant	Percent time spent in spaces that are viewable by the public	Percentage: Range (0, 100)
Percent Time Training	Elephant	Percent time spent in training sessions, including known or new behaviors	Percentage: Range (0, 100)
Percent Time Demonstration	Elephant	Percent time spent in staff-directed activities in an education or show context	Percentage: Range (0, 100)
Percent Time Relationship	Elephant	Percent time spent in staff-directed relationship building activities	Percentage: Range (0, 100)
Percent Time Play	Elephant	Percent time spent in staff-directed play activities	Percentage: Range (0, 100)
Percent Time Exercise	Elephant	Percent time spent in staff-directed exercise sessions	Percentage: Range (0, 100)
Percent Time Managed	Elephant	Percent of time an elephant spent in staff-directed activities, including exercise, husbandry, training time, play, relationship sessions, and demonstrations	Percentage: Range (0, 100)
Percent Time Independent	Elephant	Percent of time spent outside of staff-directed activities, including non-managed time on and off exhibit	Percentage: Range (0, 100)
Percent Time Other	Elephant	Percent time spent in other non-defined staff-directed activities	Percentage: Range (0, 100)
Enrichment Diversity*	Zoo	Shannon-Weaver index of the number of enrichment types and frequency with which they were provided	Continuous: Range (0 [one item used all the time], 3.4 [equal frequency use across all 30 items])
Enrichment Program*	Zoo	Standardized Factor Score created using a *polychoric* Principal Components Analysis to examine the frequency of use of the different components of an enrichment program	Continuous: Range (-2.17, 2.06) (increasing positive scores represent greater use of goal setting, documentation, evaluation, readjustment by enrichment programs)
Feed Day	Zoo	Number of feedings during the day	Count: (1, 20)
Feed Night	Zoo	Number of feedings during the night	Count: (0, 12)
Feed Total	Zoo	Number of feedings during the day and night	Count: (1, 32)
Feeding Predictability	Zoo	The predictability of feeding activities	Categorical: (1 [predictable: feeding times consistent from day to day], 2 [semi-predictable: feeding times intentionally varied by up to 60% from day to day], and 3 [unpredictable: feeding times are not scheduled and occur randomly])
Feed Diversity*	Zoo	Shannon-Weaver index of the number of feeding types and frequency with which each type was provided	Continuous: Range (0 [one type of feeding always used], 1.8 [equal frequency use across all 6 types])
Spread*	Zoo	Proportion of all feedings where food was spread through the exhibit	Continuous: Range (0, 1)
Alternate Feeding Types*	Zoo	Proportion of all feedings where food was presented in a foraging device, hidden, or hung above the exhibit.	Continuous: Range (0, 1)
Exercise Week	Elephant	Number of hours spent exercising each week including walking, stretching, and swimming	Categorical: Range (0 [<1 hour per week], 7 [>14 hours per week])
Walking Week	Elephant	Number of hours spent walking each week	Categorical: Range (0 [<1 hour per week], 7 [>14 hours per week])
Exercise Diversity*	Elephant	Shannon-Weaver index of the number of exercise types and the frequency with which each type was used	Continuous: Range (0 [one type of exercise is always used], 2.1 [equal frequency use across all 8 types])
Guide Exposure Score	Elephant	Evaluates whether an elephant lived at a facility with guides on site	Binary: Range (0 [elephant lived at an institution that does not have guides], 1 [elephant lived at an institution that has guides])
Percent Guide Interaction Time	Elephant	Percent time spent engaged with or overseen by trainers who had a guide on their person	Categorical: Range (1 [a guide was used during 1%-9% elephant-staff interactions], 11 [a guide was used in 100% of elephant-staff interactions])

*Variables calculated from raw data. All other variables describe raw data.

**Table 4:** Variables created from the Training Techniques Survey.

Variable Name	Unit of Analysis	Description	Data Type and Possible Range (min, max)
Training Item Score*	Elephant	Frequency with which an elephant experienced each of the 19 training techniques	Categorical (half integer scale): Range (1 [never used], 5 [very frequently used])
Rewarding Stimuli Techniques Score*	Elephant	Proportion of training experiences that involve the provision or removal of rewarding stimuli	Categorical: Range (1 [only aversive stimuli] to 9 [only rewarding stimuli], with 5 representing equal experience of both rewarding and

*Variables calculated from raw data. All other variables describe raw data.

#### Management Schedules

All management schedule variables were derived from the Management Survey. Managers completed a management schedule for each elephant by estimating the average percentage of the daytime hours (zoo operating hours) per month that the individual spent in various contexts. The contexts were: exercise, husbandry (including footwork, baths, veterinary care), training known (practicing known behaviors), training new (learning new behaviors), training mixed (mixed sessions of known and new behaviors), play and relationship sessions (interactions with elephant outside of training or husbandry activities), public demonstrations (staff-directed activities in education or show setting), on exhibit (not interacting with staff), off exhibit (not interacting with staff) and other (not specified). Data were confirmed upon entry to sum to 100%.

Variables for analysis were created by summing percent time across groups of behaviors as follows: Percent Time Training = sum of training known, training new, and training mixed; Percent Time Managed = sum of exercise, husbandry, Training Time, play, relationship sessions and demonstrations; Percent Time Independent = sum Percent Time On and Percent Time Off Exhibit.

#### Environmental Enrichment

All environmental enrichment variables were derived from the Management Survey. Managers indicated the percentage of days in the year the elephants had access to 30 different types of enrichment or exhibit features. They also reported how often their staff used the enrichment program components based on the SPIDER framework (*e*.*g*., setting goals, scheduling activities, documentation, evaluation, and program readjustment). We used these data to generate two enrichment variables, Enrichment Diversity and Enrichment Program, both of which provide composite descriptions of enrichment program implementation and structure. We used the Shannon-Weaver diversity index [57] to create the Enrichment Diversity score (as well as feeding and exercise. See method sections below). This index is most commonly used to characterize ecosystem communities, and scores increase as the number of species sampled rises and the abundance of these species become more even [57]. Thus, Enrichment Diversity scores characterize the variety of enrichment types utilized as well as the relative frequency with which enrichment types were presented. High Diversity Scores indicate equal and frequent use of all enrichment types, while low diversity scores indicate infrequent use of enrichment in general or reliance on frequent use of only a few enrichment types. The Enrichment Diversity scores we calculated had a possible score range of 0 (one type of enrichment always used) to 3.4 (equal frequency use across all 30 enrichment items). We used principal components factor analysis with a *polychoric* matrix (to account for the ordinal data) [58] to develop a factor matrix describing enrichment program component use. Factor loadings were retained using the proportion of variance method, and these loadings were used to calculate the standardized Enrichment Program factor scores.

#### Feeding

Feeding questions addressed the various ways food was presented to the elephants at each zoo. Managers reported the number of feeding events offered to their elephants during specific time frames (Feed Day, Feed Night, Feed Total) and the frequency with which various feeding methods were used (*e*.*g*., piled on ground, spread through exhibit, presented in an open container, suspended, presented in a foraging device, and hidden). Managers also rated the predictability of their feeding activities on a three-point scale ranging from “predictable” to “unpredictable” (Feeding Predictability). We further processed the feeding methods data to generate three synthesized variables: Feeding Diversity, Spread, and Alternative Feeding Types. The Shannon-Weaver Index was used to derive the Feeding Diversity score, with a possible range of 0 (one type of feeding always used) to 1.8 (equal frequency use of 6 feeding types). Two variables measured the use of particular types of feeding methods: Spread (the proportion of feedings where food was spread through the exhibit) and Alternative Feeding Types (the proportion of feedings where food was hidden, hung up, or presented in a foraging device).

#### Exercise

Exercise questions addressed the percentage of time individual elephants spent engaged in staff-directed exercise methods [*e*.*g*., A to Bs (directed walking from point A to point B, repeated as needed), calisthenics (*e*.*g*., climbing up and down blocks and lifting objects), intervals, slow walks, strengthening exercises, stretching, swimming, and water walking], and the number of hours per week individual elephants engaged in directed walks (Walking Week) and general exercise (Exercise Week). Walk Week and Exercise Week were categorical variables on a seven-point scale, ranging from less than one hour per week to more than 14 hours per week. We further processed the exercise data to create the Exercise Diversity variable, a score calculated using the Shannon Weaver Index with a possible range of 0 (one type of exercise always used) to 2.1 (equal frequency use across eight exercise types).

#### Training

Guide use was determined from the Management Survey, which asked managers whether there was a guide at the facility (Guide Exposure) and the frequency with which each elephant at the zoo was exposed to a guide during staff interactions (Percent Guide Interaction).

The Training Techniques Survey asked qualified care staff to rate their use of 19 different training techniques when working with the assigned elephant(s). We used these responses to create two types of synthesized variables: Training Item Scores and a Rewarding Stimuli Techniques Score (Table 4). To calculate the Training Item scores, we averaged the staff responses on each of the 19 training techniques for every elephant and then rounded these values to the nearest half integer, forming a nine-point half-integer scale that included the following categories: Never (1), Never/Rarely (1.5), Rarely (2), Rarely/Sometimes (2.5), Sometimes (3), Sometimes/Frequently (3.5), Frequently (4), Frequently/Very Frequently (4.5), and Very Frequently (5).

To calculate the Rewarding Stimuli Techniques Score, we first categorized 12 of the 19 training techniques according to whether stimuli were added or removed, then according to the valence of the stimuli (rewarding or aversive; Table 5). The remaining seven training techniques were categorized as neutral, because they did not have a clear valence. Following categorization, the scores from techniques that involved the provision of rewarding stimuli and the removal of rewarding stimuli for each elephant were summed and divided by the sum of their scores on all techniques. We categorized these values to a nine-point scale that ranged from one, meaning that training interactions lacked the use of rewarding stimuli, to nine, meaning that they utilized rewarding stimuli exclusively.

**Table 5:** Operant conditioning theory as it applies to the training methods surveyed.

	Addition/Removal of Stimulus	Stimulus Valence	Training Method Examples
	**Add**	+	• Give food
			• Give verbal praise or use a clicker or whistle
			• Give items that the elephant likes
Method designed to increase the frequency of desired behaviors			• Pat, rub, or scratch the elephant
	**Remove**	-	• Remove stimuli that the elephant does not like, e.g., contact with a guide, shift dominant animals away
	**Add**	-	• Say “No”
			• Swat with guide or object
			• Apply gentle pressure with the guide or other object
			• Apply forceful pressure with the guide or other object
Method designed to decrease the frequency of undesired behaviors			• Show the guide without making contact
	**Remove**	+	• Remove items that the elephant likes
			• Remove attention and opportunities for reward (i.e., give a time out)
		Neutral	• Provide no response to elephant’s behavior
			• Request a different behavior
			• Do not give food reinforcement
			• Give a neutral pause for 3–5 seconds
			• Request the behavior again
Methods that may provide another opportunity to succeed			• Request the same or other behavior, aiding the elephant by encouraging approach towards a target (*e*.*g*., a pole, brush, hand, or other item)

+: Stimuli that an individual finds rewarding

-: Stimuli that an individual finds aversive. Adapted from Skinner [49]

In addition to developing these two synthesized variables, we also investigated the effect of Guide Exposure on the training experience of elephants. Mann-Whitney U (Wilcoxon Rank Sum) tests were used to determine whether Training Item and Rewarding Stimuli Techniques Scores differed between the Guide Exposure subgroups.

#### Statistical Analyses

We calculated descriptive statistics for all variables, and conducted Mann-Whitney U (Wilcoxon Rank Sum) tests to determine species and sex differences for all variables except the zoo-level variables, which could only be assessed for species effects. Where the Mann-Whitney U results indicated a species or sex difference that could be better interpreted with further analyses, we used linear regression models fitted using generalized estimating equations (GEE), which allow for the individual elephant to be used as the unit of analysis and account for the clustering of individuals within zoos [59,60]. Zoos were treated as random effects and an independent correlation structure was specified [61]. We used the forward selection approach to build the models [62] and continued to add variables until the addition no longer resulted in significant models. Interactions among the variables contained in any significant multi-variable models were assessed during the final model building stage. Microsoft Excel (Microsoft Corporation, Redmond, WA) and SAS version 9.3 (SAS Institute, Cary, NC) were used for all statistical analyses. We used regression modeling code: [PROC GENMOD, with options DIST = Normal, LINK = Identity, TYPE = Ind, and REPEATED]. For all analyses, p-values ≤ 0.05 were considered statistically significant.

## Results and Discussion

Our surveys generated a large number of variables that are important for understanding how elephants are managed in North American zoos. In the results and discussion, we will focus only on the subset of those variables that can be directly related to existing literature or have clear links to animal welfare. Although African and Asian elephants are different species and are behaviorally and physiologically sexually dimorphic, there were surprisingly few species or sex differences; those we did find are discussed further below. Results from variables not discussed in the text can be found in the appropriate section Tables.

### Response Rates

Sixty-three of seventy invited zoos (90%) submitted completed Management Surveys. Four zoos (5.7%) submitted partially completed surveys, and three (4.3%) zoos declined participation. The number of elephants included in each variable analysis ranged between 83 and 224 based on data availability and applicability. Completed Training Techniques Surveys were submitted by 62 zoos for a total of 602 surveys on 227 elephants.

### Management Schedule

We identified significant species level differences in seven of the 11 Management Schedule variables (Table 6), with the primary difference being that Asian elephants were managed for a significantly greater proportion of their day (mean = 56.9% *p*<0.001) than African elephants (mean = 48.6). During managed time, Asian elephants also spent significantly more time engaged with staff in husbandry activities (mean = 15.5%), exercise (mean = 6.8%), and play (mean = 4.0%), than did African elephants (mean = 12.2%, 4.3%, and 1.9% respectively). Conversely, African elephants spent significantly more time independent (mean = 51.4%, *p*<0.001), than did Asian elephants (mean = 43.1%). The only sex difference was that females spent more time in exercise (mean = 5.5%) than males (mean = 4.3%).

**Table 6:** Management Schedule variables for population and by species and sex. Comparisons between species and between sexes were made using the Mann-Whitney U (Wilcoxon Rank Sum) test.

						Species							Sex						
	Full Population					African			Asian				Male			Female			
	N^1^	Mean	SEM	Min	Max	N	Mean	SEM	N	Mean	SEM	*p*	N	Mean	SEM	N	Mean	SEM	*p*
*Management Schedule*																			
Percent Time Off Exhibit	189	8.0	0.7	0	40	106	7.9	1.0	83	8.2	1.0	0.069	39	9.7	2.0	150	7.6	0.8	0.694
Percent Time On Exhibit	206	40.6	1.4	0	80	121	44.5	1.6	85	35.1	2.3	<0.001*	41	41.1	3.0	165	40.5	1.6	0.94
Percent Time Training	206	19.4	0.6	3	40	121	19.8	0.8	85	18.8	1.0	0.386	41	20.8	1.4	165	19.1	0.7	0.193
Percent Time Demonstrations	200	4.4	0.3	0	20	117	4.2	0.4	83	4.7	0.4	0.132	40	4.1	0.6	160	4.4	0.3	0.657
Percent Time Relationship	186	7.4	0.7	0	51	106	7.7	1.1	80	6.9	0.9	0.163	40	6.3	1.4	146	7.7	0.9	0.375
Percent Time Husbandry	206	13.5	0.5	1	35	121	12.2	0.5	85	15.5	0.9	0.003*	41	11.9	1.0	165	13.9	0.5	0.067
Percent Time Play	184	2.9	0.3	0	15	101	1.9	0.3	83	4.0	0.4	<0.001*	38	2.4	0.5	146	3.0	0.3	0.617
Percent Time Exercise	201	5.3	0.4	0	20	118	4.3	0.5	83	6.8	0.5	<0.001*	41	4.3	0.9	160	5.5	0.4	0.042*
Percent Time Managed	206	52.0	1.4	13	99	121	48.6	1.7	85	56.9	2.1	<0.001*	41	49.7	2.9	165	52.6	1.5	0.425
Percent Time Independent	206	48.0	1.4	0	87	121	51.4	1.7	85	43.1	2.1	<0.001*	41	50.3	2.9	165	47.4	1.5	0.425
Percent Time Other	83	1.3	0.4	0	15	57	0.3	0.1	26	3.5	1.0	<0.001*	14	1.0	0.8	69	1.3	0.4	0.87

^1^Ns vary based on data availability and on the fact that some management practices are not applicable to individual elephants

*p significant at <0.05. Zoos that housed both Asian and African elephant species were removed from the species-level analysis (N = 5 zoos).

Linear regression analysis of Percent Time Managed demonstrated that age and species contributed to the amount of time an elephant spent in managed activities (Tables 7 and 8). Managed time increased by 20.2% for every year of age, and Asian elephants were 6.2 times more likely to spend more time in managed activities than African elephants. There were no significant interactions between age and species, indicating that increases in age influence managed time at the same rate for the two species. Age is a known contributor to foot pathology in both species [13,33], and it may be that additional time is being spent in foot care with older elephants to mitigate risks. However, since both age and species are main effects in this model, we can infer that there is something about being an Asian elephant (other than the fact that Asians in this population are older than Africans [7]) driving species differences in managed time. One possibility is that more managed time is being allocated to address musculoskeletal health issues [33,34] or stereotypic behaviors [9] which are more prevalent in Asian elephants. Since research in humans [37,38] and other species (rodents [40,41], dogs [44]) demonstrates that physical activity can reduce health risks associated with musculoskeletal health issues, managers may be engaging older and Asian elephants in active managed activities (*e*.*g*., exercise and play) in an effort to mitigate these problems. Similarly, managers may be engaging older and Asian elephants in more foot care related husbandry activities in an effort to treat existing foot pathologies.

**Table 7:** Demographic variables tested for association with Percent Time Managed and statistics associated with the univariate linear regression models.

Hyp^1^	Variable	Reference	β-coefficient	N	p*
**+**	**Age**	None	0.261	219	0.003*
**+**	**Species**	Ref = African		131	
		Asian	7.788	88	0.006*

*Variable was retained for the model building process when *p*<0.05.

^1^Hypothesized direction of effect

**Table 8:** Linear regression model for demographic variables associated with Percent Time Managed (N = 219, QIC = 221.4).

Variable	β-coefficient	Standard Error	95% Confidence Limits		p*
**Intercept**	43.355	2.730	38.005	48.706	<0.001*
**Age**	0.202	0.090	0.026	0.378	0.024*
**Species (African)**	0				
**Species (Asian)**	6.230	2.900	0.546	11.915	0.031*

*Significant when *p*<0.05

It should be noted that the night period was not included in any of the Management Schedule analyses. In most cases, elephants are independent for the entire night which, for this population, averaged between 8–18 hours depending on the season, with a modal value of 14 hours in both the summer and winter [15].

### Environmental Enrichment

**Fig 1:**
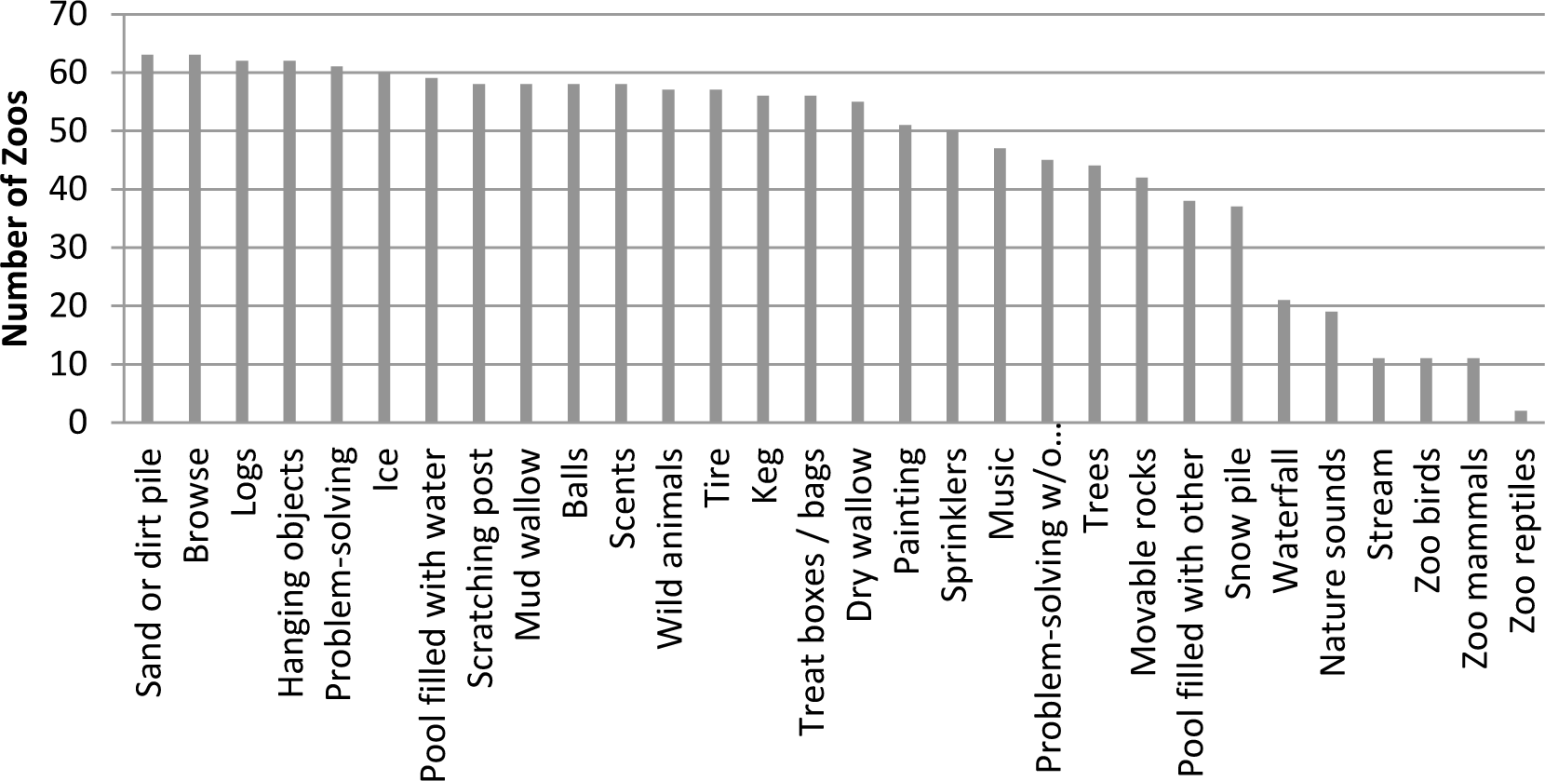
The number of zoos that provided each of the 30 enrichment types.

**Fig 2:**
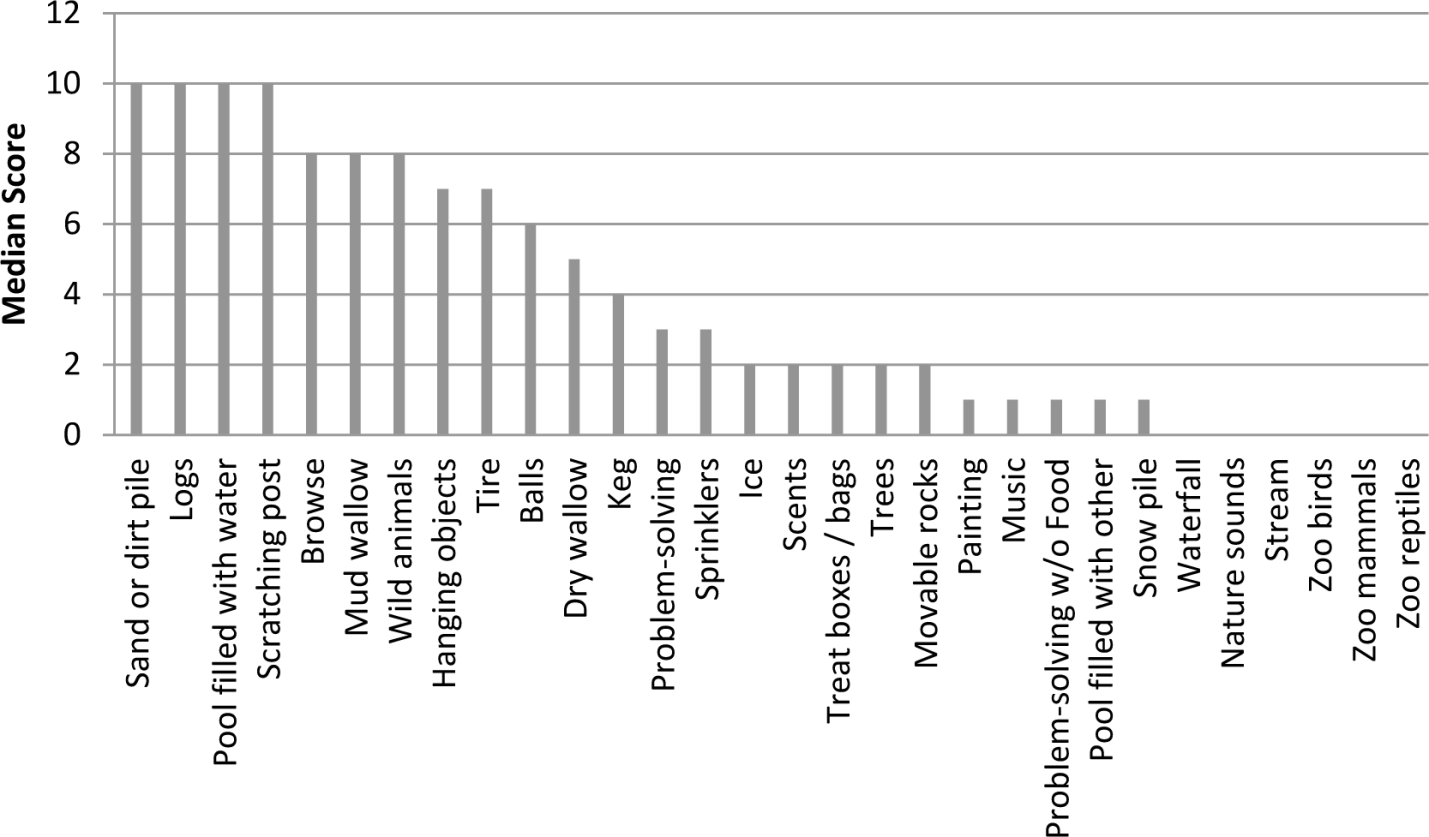
Median-enrichment and exhibit-feature use scores of the 63 zoos. Each categorical scale score shows ranges in 10% increments (*e*.*g*., 1 = 1–9%, 2 = 10–19%, etc.). Scores of 0 represent no use and scores of 11 represent use 100% of the time.

**Fig 3:**
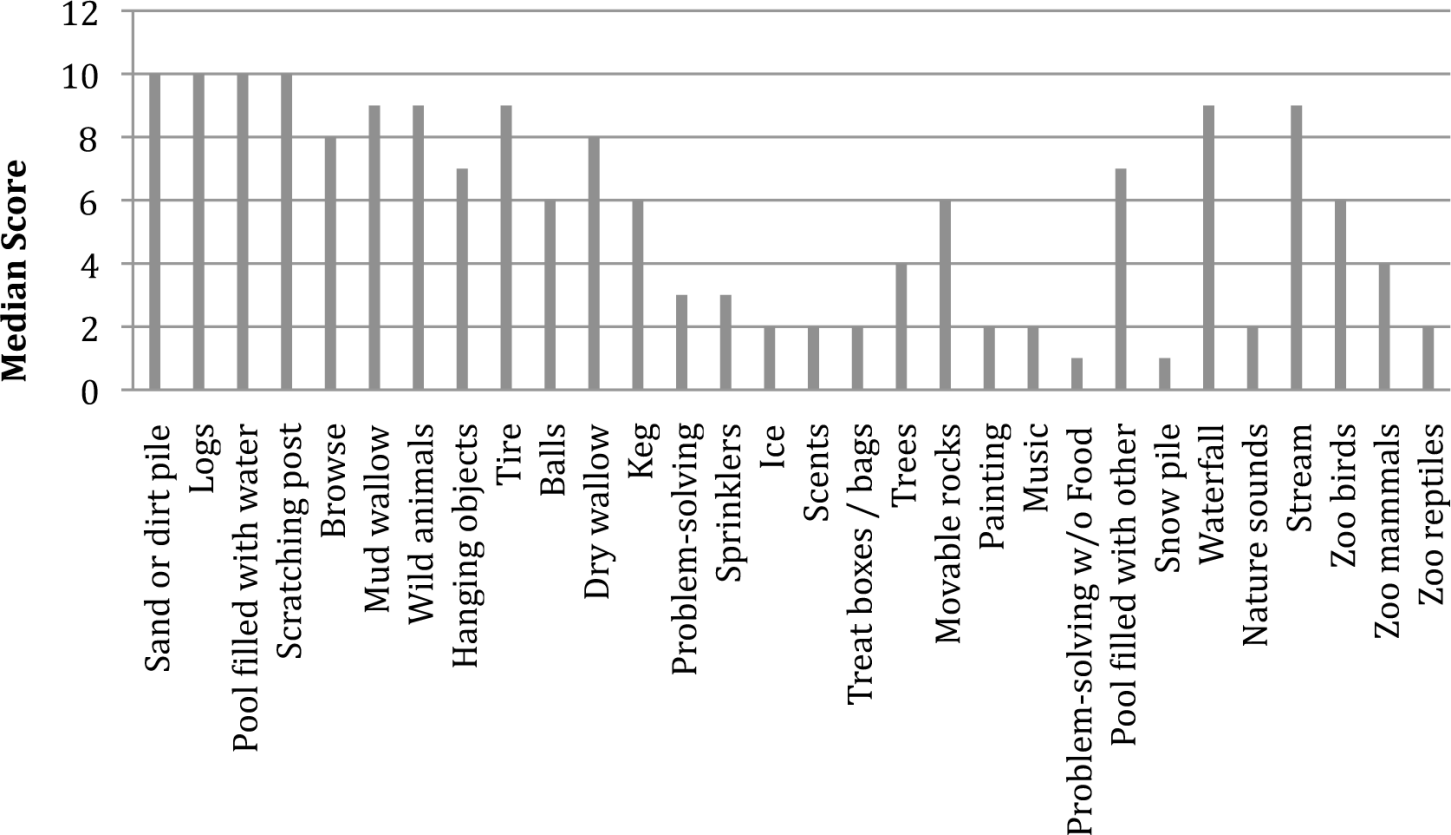
Median-enrichment and exhibit-feature use scores from zoos that used these methods. Each categorical scale score shows ranges in 10% increments (*e*.*g*., 1 = 1–9%, 2 = 10–19%, etc.). Scores of 0 represent no use and scores of 11 represent use 100% of the time.

All sixty-three zoos provided their elephants with enrichment (Table 9), (median = 73% of enrichment options included in our survey, max 97%, min 43%). Of these, the most common types were dirt piles and browse, which were used to some degree by all zoos (Fig 1). With respect to frequency of provision, most zoos provided dirt piles, pools, logs, and scratching posts nearly every day (median = 10; available on 90–99% of days) (Figs 2 and 3). These features could be important to facilitate self-maintenance behaviors and maintain skin condition and, as such, are integrated into enrichment programs as permanent or semi-permanent exhibit features. Our data also show that while problem-solving opportunities were components of most enrichment programs (97% of zoos), they were provided infrequently (problem-solving opportunities with food rewards median = 3; problem-solving opportunities with non-food rewards median = 1). Taken together these findings are consistent with a survey-based study that examined enrichment practices for mammals at 60 Oceanian, North American, and European zoos. Hoy et al. [63] found that permanent or semi-permanent exhibit features were perceived by staff to be the most important types of enrichment, and that enrichment types that required considerable time to prepare or were difficult to set up were provided less frequently. Logistical problems associated with time or preparation difficulty may explain why our data also show lower frequencies of use of problem solving methods, as they may need to be tailored to individual animals and regularly updated to ensure the animals continue to use their cognitive and behavioral skills in novel ways [20]. Since problem-solving requires animals to use cognitive and behavioral skills to exert control over their environment and can be self-reinforcing [20,64], we had hoped to test the effects of problem solving enrichment in the subsequent welfare models. However, we were unable to do so, since the frequency of use was so low.

**Table 9:** Enrichment variables for the full population and by species. Comparisons between species and between sexes were made using the Mann-Whitney U (Wilcoxon Rank Sum) test.

						Species						
	Full Population					African			Asian			
	N^1^	Mean	SEM	Min	Max	N	Mean	SEM	N	Mean	SEM	*p*
*Enrichment*												
Enrichment Diversity^A^	63	2.9	0	2.3	3.3	33	2.8	0	26	2.9	0	0.111
Enrichment Program^B^	63	0	0.4	-2.2	2.1	33	0	0.2	26	0	0.2	0.897

^A^Continuous score with a possible range from 0 (one item used all the time) to 3.4 (equal frequency use across all 30 items)

^B^Continuous score with a possible range from -2.2 to 2.1 (increasing positive scores represent greater use of goal setting, documentation, evaluation, readjustment by enrichment programs)

^1^Ns vary based on data availability and on the fact that some enrichment practices are not applicable at individual zoos

*p significant at <0.05. Zoos that housed both Asian and African elephant species were removed from the species-level analysis (N = 5 zoos)

Like Hoy et al. [63] we found that scheduling enrichment with a calendar was the most frequently used enrichment program component (median = 4; frequently) derived from the SPIDER framework. The other four program components were only used sometimes (median = 3 for each component) by most zoos, resulting in mid-range Enrichment Program scores (mean = 0; on a scale from -2.2 to 2.1). Equal and integrated use of all five of these components is likely to ensure that enrichment activities continue to be stimulating for animals [27].

Enrichment Diversity scores were moderate (mean = 2.9 on a scale from 0 to 3.4), indicating that there is opportunity to increase the variety and frequency of enrichment presentation for zoo elephants. To our knowledge, only two other large scale studies have attempted to quantify enrichment complexity, but using different indices than the Shannon Weaver. One [65] used an index that places less emphasis on evenness and gives more weight to the most dominant types experienced, and thus underestimates variety[57], while the other [66] generated a novel “environmental complexity” score. Given the complex nature of enrichment programs, it is necessary that methods for quantifying this practice continue to evolve such that variability within and between programs can be robustly assessed. For example, both measures that account for how individual animals utilize enrichment items and how novelty is incorporated into enrichment provisioning are topics that could be included to strengthen assessment methods.

### Feeding

Research on elephants and other species has shown that providing animals with smaller portions of food more frequently can improve body condition (sows [67]), reduce abnormal behavior (Asian elephants, giraffe, okapi [28,68]), and increase naturalistic feeding behavior (African elephants [25]). Since elephants in the wild feed during diurnal and nocturnal periods [5,6], a pattern of smaller more frequent meals could be beneficial during the daytime and the nighttime. However, most elephants were fed only once during the nighttime (Tables 10 and 11). While we did not collect data on the timing of this feeding, other studies have shown that nocturnal feedings are often offered at the beginning of the nighttime management period [69,70]. Providing food in this manner has been linked to rapid consumption early in the evening and a linear decrease in feeding behavior as the nighttime progresses [70].

**Table 10:** Feeding variables for population and by species. Comparisons between species and between sexes were made using the Mann-Whitney U (Wilcoxon Rank Sum) test.

						Species						
	Full Population					African			Asian			
	N	Mean (Median)	SEM (IQR)	Min	Max	N	Mean (Median)	SEM (IQR)	N	Mean (Median)	SEM (IQR)	*p*
*Feeding*												
Feed Day^A^	64	6.0	0.5	1	20	37	6.1	0.7	27	6	0.8	0.708
Feed Night^A^	64	1.2	0.2	0	12	37	0.8	0.2	27	1.6	0.5	0.236
Feed Total^A^	64	7.2	0.6	2	32	37	6.9	0.7	27	7.7	1.2	0.935
Feeding Predictability^B^	64	(2)	(0)	1	3	37	(2)	(0)	27	(2)	(0)	0.867
Feeding Diversity^C^	64	1.3	0	0.3	1.8	37	1.4	0	27	1.3	0.1	0.959
Spread^D^	64	0.2	0	0	0.7	37	0.3	0	27	0.2	0.0	0.106
Alternate Feeding Types^D^	64	0.4	0	0.1	0.9	34	0.4	0	30	0.4	0.0	0.648

^A^Count of the number of feeding events in specific management periods, counts ranged between 0–32

^B^Categorical score with a possible range from 1 (predictable feeding times) to 3 (unpredictable feeding times)

^C^Continuous score with a possible range from 0 (one type of feeding always used) to 1.8 (equal frequency use across all 6 food distribution types)

^D^Frequency ranging from 0 to 1

*p significant at <0.05. Zoos that housed both Asian and African elephant species were removed from the species-level analysis (N = 5 zoos)

**Table 11:** Feeding Predictability frequencies of scores.

Feed Predictability	Number of Zoos
1—Predictable	13
2—Semi-Predictable	38
3—Not Predictable	13

**Fig 4:**
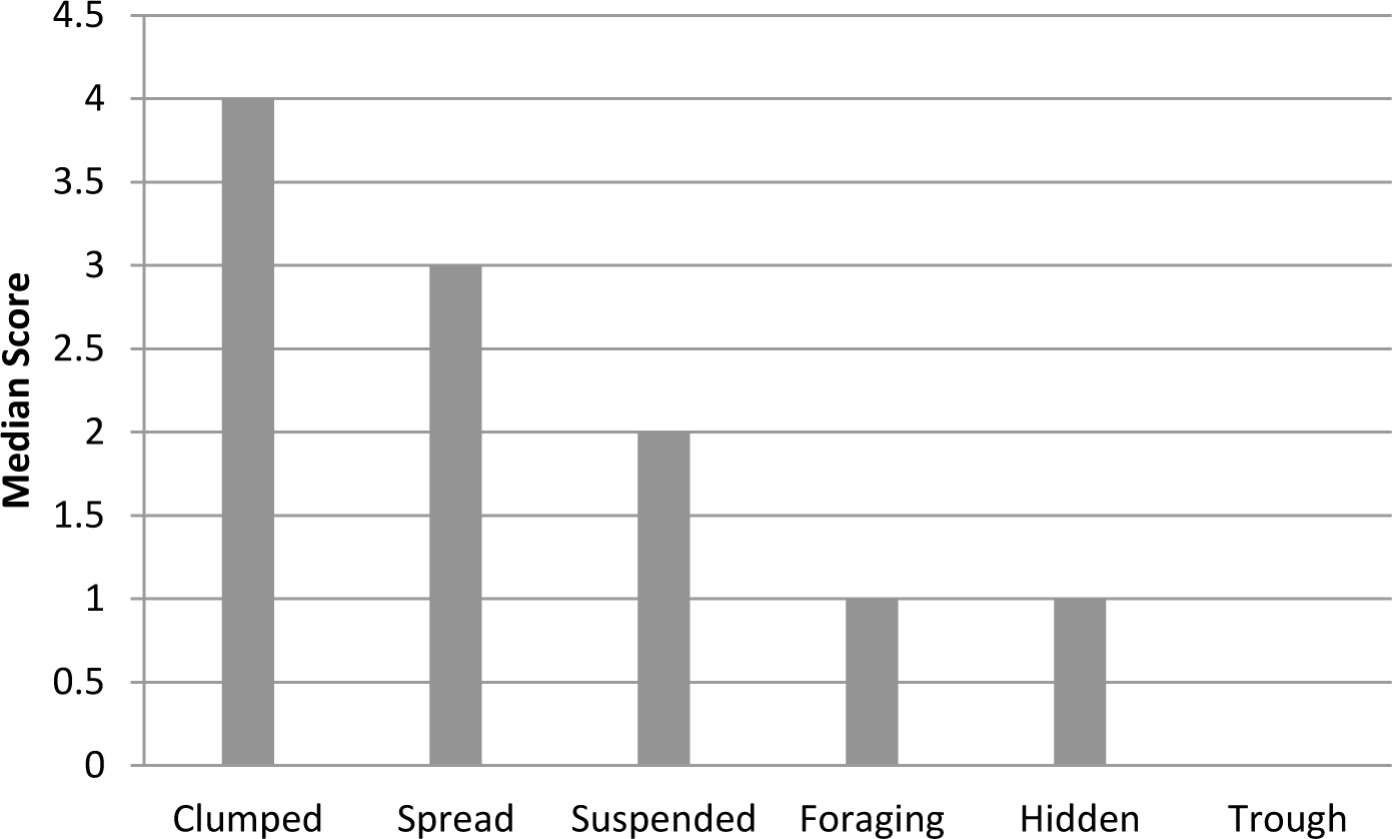
Median scores for feed presentation methods from 64 zoos. Each categorical scale score represents use ranges in 10% increments (*e*.*g*., 1 = 1–9%, 2 = 10–19%). Scores of 0 represent no use and scores of 11 represent use 100% of the time. Categories included clumped [food placed in piles composed of multiple flakes of hay, browse items, or pellet], spread [food distributed through the exhibit], suspended [food suspended by rope, in a bag, open sided barrel, etc.], foraging [food provisioned in a feeding apparatus], hidden [food hidden around the exhibit], trough [food place in an open trough].

**Fig 5:**
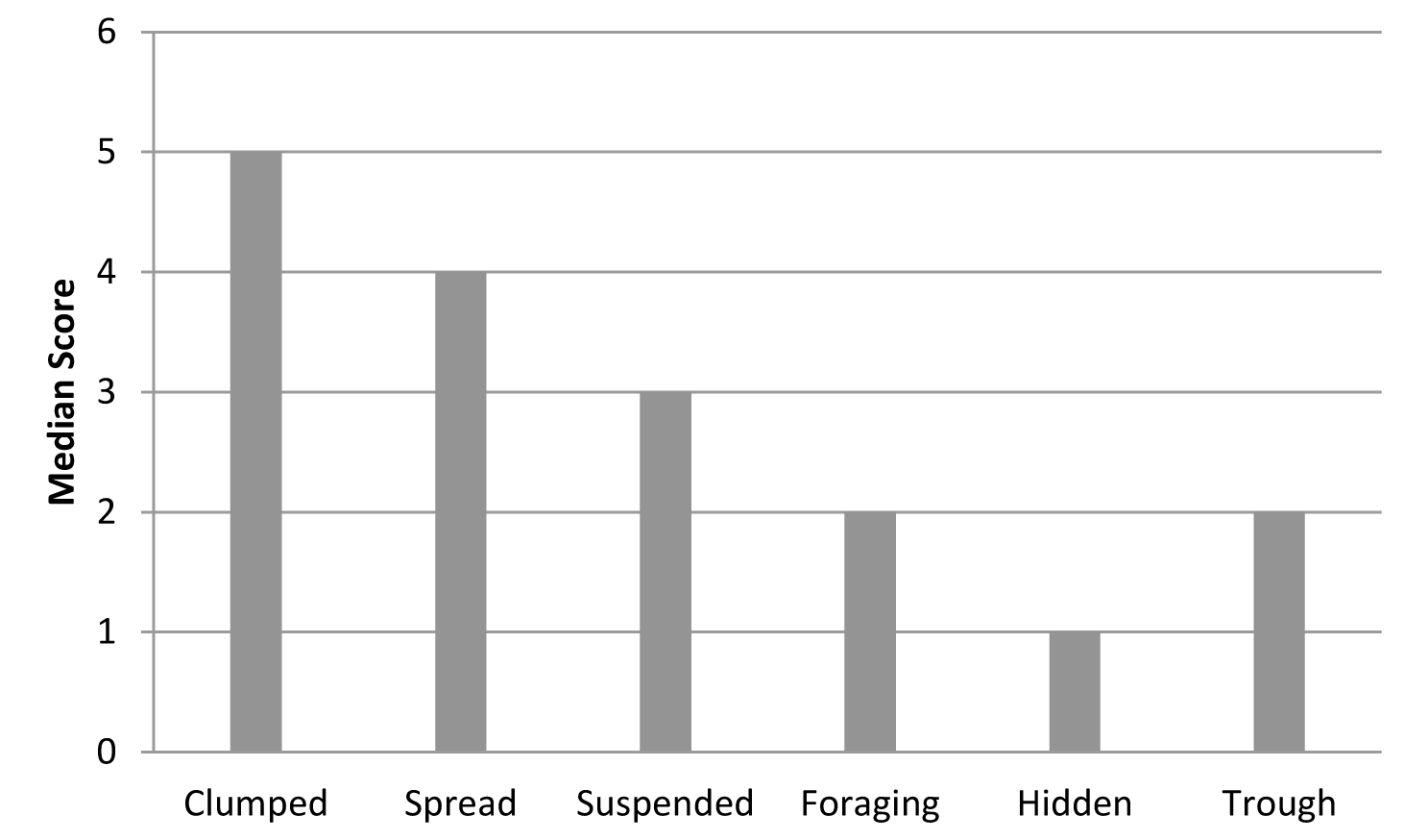
Median scores for feed presentation methods from zoos that used each method. Each categorical scale score represents use ranges in 10% increments (*e*.*g*., 1 = 1–9%, 2 = 10–19%). Scores of 0 represent no use and scores of 11 represent use 100% of the time. Categories included clumped [food placed in piles composed of multiple flakes of hay, browse items, or pellet] (66 zoos used this method), spread [food distributed through the exhibit] (66 zoos used this method), suspended [food suspended by rope, in a bag, open sided barrel, etc.] (63 zoos used this method), foraging [food provisioned in a feeding apparatus], hidden [food hidden around the exhibit] (59 zoos used this method), trough [food placed in an open trough] (29 zoos used this method).

Nearly half (47.5%) of all zoos feed their elephants on a predictable schedule, with food most commonly presented in clumps (*i*.*e*., piles composed of multiple flakes of hay, browse items, or pellet) on the ground (median = 4; 30–39% of feedings included this method) (Figs 4 and 5). The low average Spread scores (mean = 0.2, on a scale from 0 to 1) and Feeding Diversity scores (mean = 1.3 on a scale ranging from 0 to 1.8) also indicate that food is typically provided in concentrated locations with a moderate amount of variety in food presentation. Taken together, these results indicate that many elephants are fed on temporally predictable schedules with high spatial predictability and only moderate feeding method variation. Relying on temporally and spatially predictable feeding programs is potentially problematic. Animals are capable of anticipating the arrival of predictable events, even in the absence of external cues [71], and this ability is hypothesized to play an important role in stimulating appetitive searching behaviors [32]. However when food is presented in a temporally and spatially predictable manner, these appetitive behaviors can become dissociated from their original function and develop into stereotypic behaviors [32]. In some species, food-associated anticipatory stereotypies can be disrupted and reduced by providing animals with temporally and spatially unpredictable feeding events [31], and it seems possible the same may apply to elephants.

### Exercise

**Fig 6:**
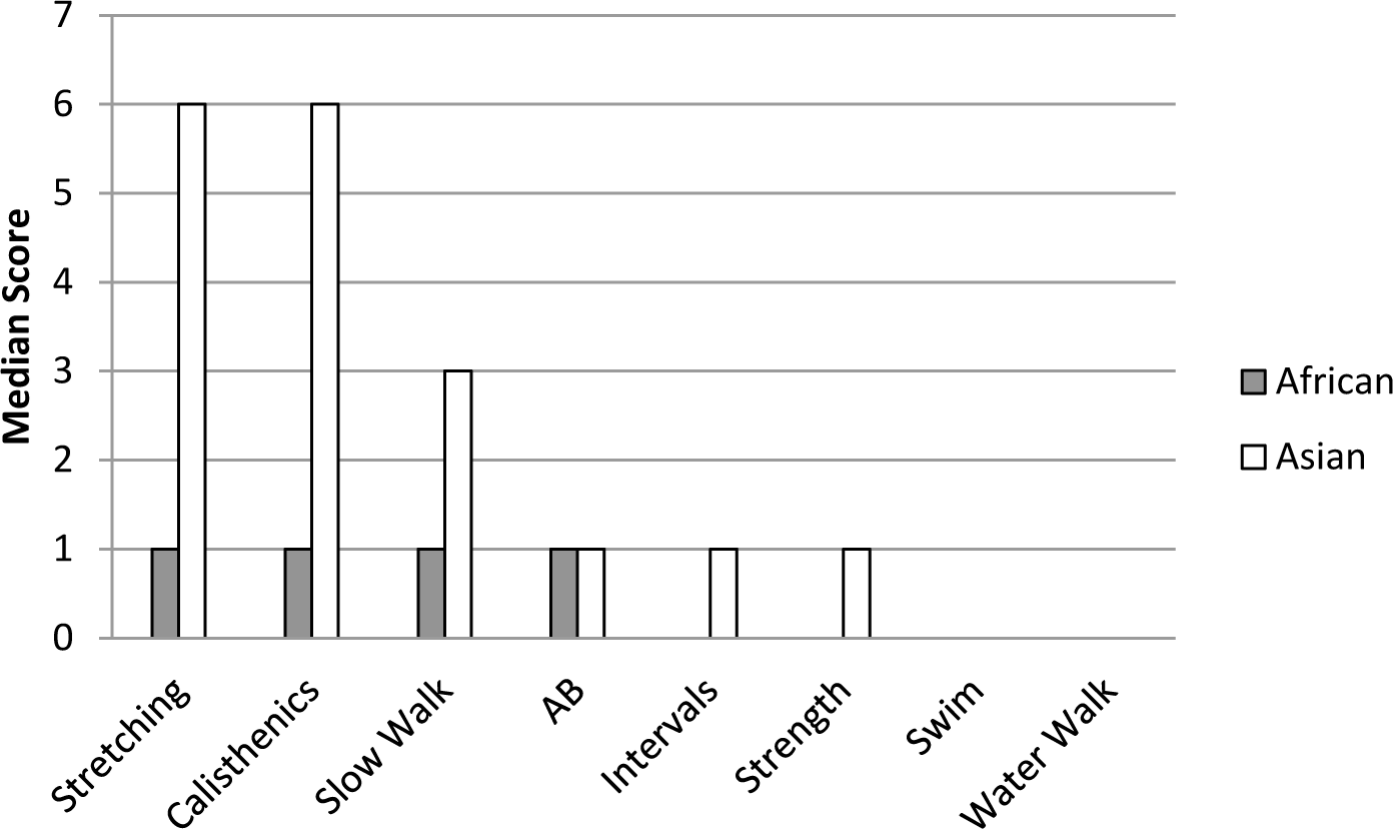
Median scores for exercise methods used for African and Asian elephants. Each categorical scale score represents use ranges in 10% increments (*e*.*g*., 1 = 1–9%, 2 = 10–19%, etc.). Scores of 0 represent no use and scores of 11 represent use 100% of the time. Categories included: stretching, calisthenics (*e*.*g*., climbing up and down blocks or lifting objects), A to Bs (directed walking from point A to point B, repeated as needed), intervals (directed walking at different rates), slow walking, strength building (*e*.*g*., lifting or pulling heavy objects), swimming, and water walking (directed walking in shallow water).

Asian elephants had higher scores on all exercise variables than African elephants (Tables 12 and 13), confirming and expanding upon the management schedule findings. African elephants were rarely given any exercise (Fig 6), while elephant managers typically engaged Asian elephants in a variety of types of exercise, including stretching, calisthenics, and slow walks. Considering that the AZA’s exercise program requirement is intended to mitigate complications associated with degenerative bone disease and obesity [3], it is interesting that aerobic exercise types are not used more frequently. Aerobic exercise has been shown in humans [35,36] and other species (rodents [39,42], dogs [43,45], swine [46]) to reduce the risk of obesity and, when combined with strength building exercises, is also important for treating and reducing the risk of bone and joint diseases (humans [37,38], rodents [40,41], dogs [44]).

**Table 12:** Exercise variables for population and by species and sex. Comparisons between species and between sexes were made using the Mann-Whitney U (Wilcoxon Rank Sum) test.

						Species							Sex						
	Full Population					African				Asian				Male			Female		
	N	Mean (Median)	SEM (IQR)	Min	Max	N	Mean (Median)	SEM (IQR)	N	Mean (Median)	SEM (IQR)	*P*	N	Mean (Median)	SEM (IQR)	N	Mean (Median)	SEM (IQR)	*p*
*Exercise*																			
Exercise Week^A^	224	(2)	(3)	1	7	133	(2)	(3)	91	(4)	(3)	<0.001*	46	(2)	(3)	178	(2)	(3)	0.087
Walking Week^A^	224	(2)	(1)	1	7	133	(1)	(1)	91	(2)	(3)	<0.001*	46	(1)	(1)	178	(2)	(1)	0.257
Exercise Diversity^B^	224	1.2	0.0	0	2	133	1	0.1	91	1.4	0.1	<0.001*	46	1.1	0.1	178	1.2	0	0.594

^A^Categorical score with a possible range from 0 (<1 hour per week) to 7 (>14 hours per week)

^B^Continuous score with a possible range from 0 (one type of exercise always used) to 2.1 (equal frequency of use across all 8 exercise types)

*p significant at <0.05. Zoos that housed both Asian and African elephant species were removed from the species-level analysis (N = 5 zoos)

**Table 13:** Exercise Week and Walk Week frequencies of scores by species.

	Exercise Week			Walk Week		
	African	Asian	All	African	Asian	All
1- < 1 hour	41	7	48	70	30	100
2–1–3 hours	53	30	83	46	34	80
3–3–5 hours	7	6	13	0	0	0
4–5–7 hours	12	12	24	6	11	17
5–7–10 hours	9	27	36	2	10	12
6 -10-14 hours	2	3	5	5	2	7
7 - >14 hours	9	6	15	4	4	8

### Training

Most of the elephants in this study were trained using a variety of methods, the majority of which involved rewarding stimuli (Rewarding Stimuli Technique Score median = 7) (Tables 14 and 15). The predominance of these techniques may reflect awareness that their use can minimize the animals’ negative states (*i*.*e*., frustration) during training and lead to more efficient individualized learning [51,72] by increasing communication clarity [51,73]. Additionally, the removal of rewarding stimuli as a means of reducing undesired behavior is less likely to cause negative affective states and/or frustration than the addition of aversive stimuli [51,52].

**Table 14:** Training variables for population and by species and sex. Comparisons between species and between sexes were made using the Mann-Whitney U (Wilcoxon Rank Sum) test.

						Species							Sex						
	Full Population					African			Asian				Male			Female			
	N^1^	Median	IQR	Min	Max	N	Median	IQR	N	Median	IQR	*p*	N	Median	IQR	N	Median	IQR	*p*
*Training *																			
Guide Exposure Score^A^	219	1	1	0	1	127	1	1	92	0	1	0.052	45	1	1	174	1	1	0.61
Percent Time Guide Interaction^B^	86	6	8	1	11	55	6	8	31	8	7	0.58	15	4	5	71	8	8	0.02
Rewarding Stimuli Techniques Score^C^	227	7	1	5	9	121	7	1	106	7	1	0.541	43	7	1	184	7	1	0.037*

^A^Binary score with a possible range from 0 (elephant lived at an institution that does not have guides) to 1 (elephant lived at an institution that has guides)

^B^Categorical score with a possible range from 1to 11, each categorical scale score (1–10) represents guide use ranges in 10% increments (*e*.*g*., 1 = 1–9%, 2 = 10–19% of elephant-staff interactions etc.) A score of 11 indicates that a guide was used in 100% of elephant-staff interactions)

^C^Categorical score with a possible range from 1 (never trained with rewarding stimuli) to 9 (very frequently trained with rewarding stimuli)

^1^Ns vary based on data availability and on the fact that some management practices are not applicable to individual elephants

*p significant at <0.05. Zoos that housed both Asian and African elephant species were removed from the species-level analysis (N = 5 zoos)

**Table 15:** Rewarding Stimuli Training Technique Score frequencies by sex.

RPRNP	Male	Female	All
1	0	0	0
2	0	0	0
3	0	0	0
4	0	0	0
5	0	12	12
6	4	29	33
7	18	76	94
8	20	66	86
9	1	1	2

**Fig 7:**
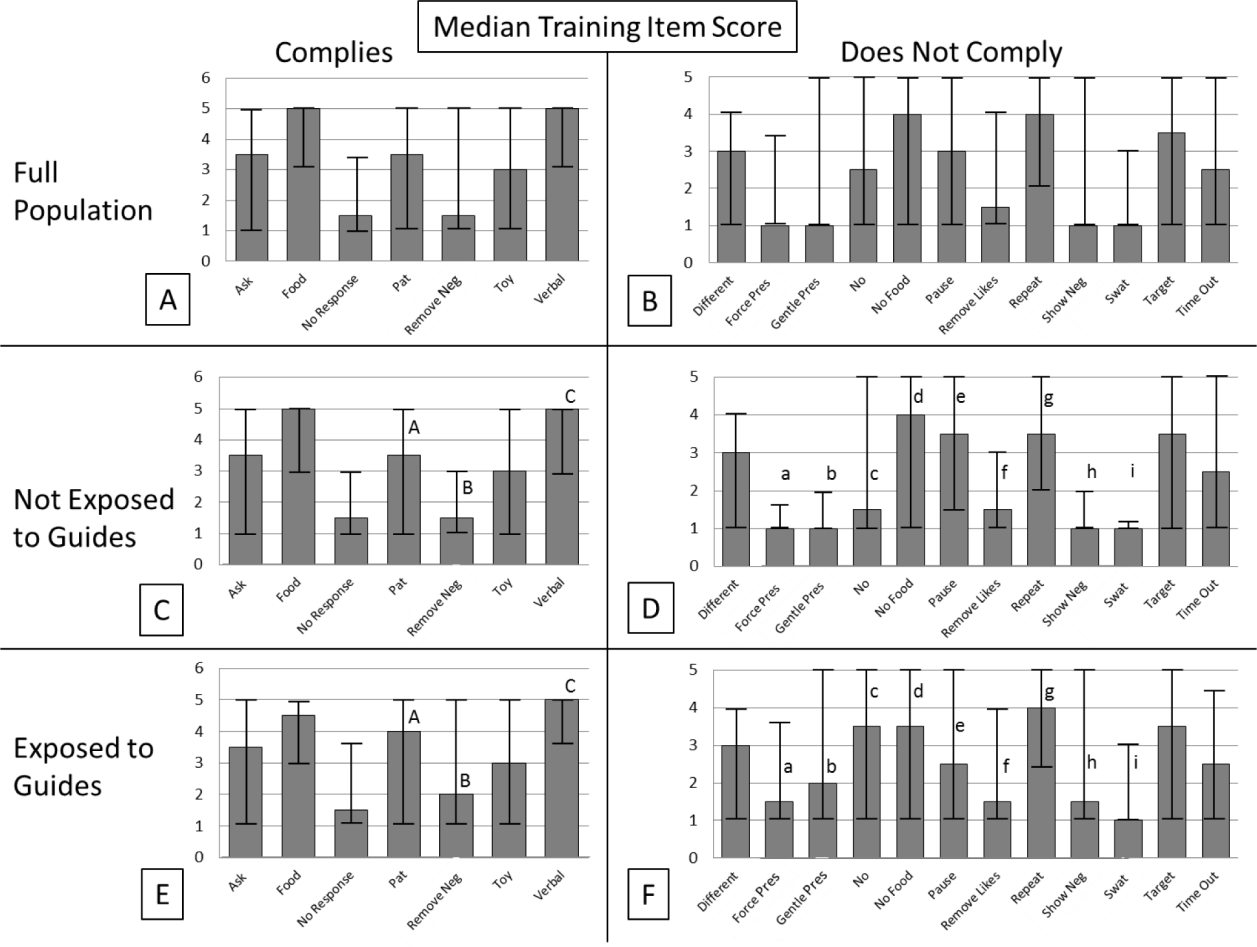
**Median Training Item scores at the level of the full population (A-B) for elephants with Guide Exposure scores equal to zero (C-D), and for elephants with Guide Exposure scores equal to one (E-F).** Training Item scores were sorted according to whether an elephant complied with a request. Scores range in half integer values between 1 (never) and 5 (very frequently) and bars represent score ranges. Significant differences in Training Item scores by guide exposure and response type are lettered (*p*<0.05). Matching case-sensitive letters indicate a significant difference for specific median training item scores between guide exposure groups. Training techniques experienced by elephants when compliant (those designed to increase the frequency of desired behavior) are left unshaded. Training techniques experienced by elephants when non-compliant (those designed to decrease the frequency of undesired behavior) are shaded to help distinguish between the different stimuli involved when addressing non-compliant behavior. Neutral items are shaded in light grey. Rewarding items are shaded with hash marks. Verbally-aversive items are shaded in medium grey. Visually-aversive items are shaded with a wave pattern. Physically-aversive items are shaded in dark grey.

**Fig 8:**
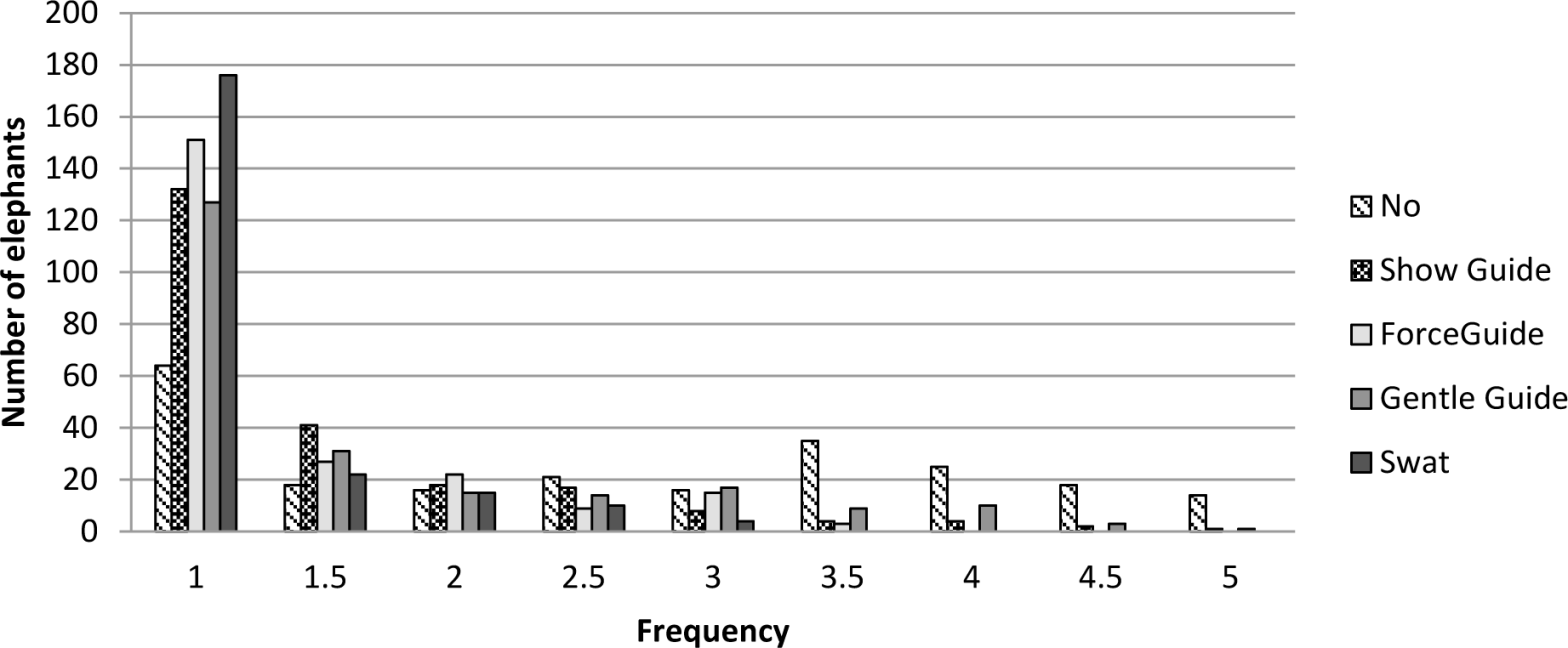
The frequency with which elephants experience each of the various aversive training techniques when non-compliant. Frequency scores range between 1 (never) and 5 (very frequently). Verbal and visual stimuli are patterned, and physical stimuli are shaded in grey tones.

Providing food rewards and positive verbal stimuli were the most common techniques utilized when elephants complied with a request (Fig 7A), and neutral techniques such as repeating the request for a behavior and not giving a food reward were most frequently used when elephants did not comply with a request (Fig 7B). Saying “No” was the aversive stimulus used most often to reduce the frequency of undesired behavior [experienced by 72% of elephants, median frequency = 3.5 (sometimes/frequently)], while aversive techniques involving physical contact were experienced by 44% percent of elephants, with a median frequency of 2 (rarely) (Fig 8).

When we subdivided the population according to Guide Exposure, we found that 81 elephants were housed at 27 zoos that did not keep guides on their premises, while 138 elephants were housed at 38 zoos with guides onsite (Table 14). Of these 138 elephants, however, 52 were never trained with a guide (Table 16), which indicates that guides were not used for routine management of these elephants but may have been kept onsite for emergencies. At nine zoos, we found that some elephants were trained with guides, while others were not. This within-zoo variation highlights the need to assess training at the level of the individual elephant rather than the herd. Analyses of Training Item scores by Guide Exposure revealed several significant differences (Fig 7C–7F). These differences showed a general trend for elephants that were exposed to guides to experience techniques that involved the removal (*e*.*g*., remove negative) or addition of aversive stimuli (*e*.*g*., gentle pressure, “No,” showing an aversive stimulus) at a significantly higher frequency than elephants that were not exposed to guides. Accordingly, Rewarding Stimuli Technique Scores were lower for elephants with guide exposure (median_(GE = 1)_ = 7, median_(GE = 0)_ = 8; U = 11,474.5, p<0.05). All of these findings demonstrate that exposure to guides coincides with more frequent use of aversive stimuli in training interactions. Interestingly, the converse appears not to be true, since 97.5% of all elephants at zoos where guides were not on site experienced aversive stimuli during training when non-compliant. Therefore, our study shows that while the frequency with which these techniques are utilized is higher for animals that are trained with guides, the absence of guides in the training environments does not guarantee that techniques that have been shown to evoke negative emotional states [51,53] or behavioral responses [54,74] in humans and other animals are not being used. However, it is also important to note that across the whole population, these types of techniques are used with much less frequency [median = 1.5 (never/rarely)] than techniques involving rewarding stimuli [median = 3.5 (sometimes/frequently)] and neutral techniques [median = 3.5 (sometimes/frequently)].

**Table 16:** The number of elephants experiencing each Percent Time Guide Interaction score interval.

Percent of Interactions with Guide	Number of Elephants
0—Guide Never Used	52
1 –Guide used in 1–9% of interactions	17
2—Guide used in 10–19% of interactions	3
3- Guide used in 20–29% of interactions	8
4- Guide used in 30–390% of interactions	6
5- Guide used in 40–49% of interactions	1
6- Guide used in 50–59% of interactions	10
7- Guide used in 60–69% of interactions	0
8- Guide used in 70–79% of interactions	3
9- Guide used in 80–89% of interactions	0
10- Guide used in 90–99% of interactions	5
11—Guide used in 100% of interactions	33

The few studies that have attempted to evaluate the welfare effects of training categorize methods according to whether they rely entirely on the addition of rewarding stimuli, aversive stimuli, or a mix of methodologies (techniques involving the removal of rewarding stimuli are nearly always excluded from examination) without quantification of the relative proportion with which rewarding stimuli or aversive stimuli are experienced (dogs [54,74] horses [75]). Our study demonstrates that elephant training (and likely the training of many other zoo species) is practiced along a continuum. Thus, categorizing training without quantifying the degree to which different methods are used, or classifying training programs simply based on the presence or absence of guides, may mask valuable information about the variability in training experiences of individual elephants. It is also possible that, within the operant conditioning framework, different techniques have different effects on welfare. The Rewarding Stimuli Techniques Score and Training Item scores we developed in this study are the first to quantify the continuum of experiences of animals with respect to the operant conditioning framework, including those trained with mixed conditioning methods. The challenge from here forward is to test the association between these types of operationalized training variables and behavioral or physiological outcomes, such that training protocols may be optimized to support positive welfare.

## Conclusion

Assessing the welfare of zoo animals requires detailed descriptions of their physical and social environments as well as the day-to-day care practices they experience. Remarkably few studies have examined the practices used in managing zoo animals, and ours is the first multi-institutional study to do so in a systematic manner. Our methodology provides a model that could be applied to assessments of management practices for a variety of zoo-housed species. In particular, our methods for assessing enrichment program quality and diversity and the utilization of training techniques should be broadly applicable. In addition to describing animal populations of interest, a major strength of the types of variables we have synthesized is that they can be readily used as resource based measures to assess and improve welfare [76]. For example, we and our colleagues have found that the management factors described in this paper play an important role in predicting stereotypic behavior rates, walking rates, female reproductive physiology, and body condition in zoo elephants. More specifically, stereotypic behavior rates were negatively associated with Percent Time Managed [9], distances walked were positively associated with Feeding Diversity scores and an unpredictable Feed Schedule [10]. Also, the likelihood of a female African elephant having normal ovarian cyclicity was improved with her Enrichment Diversity score [12]; normal prolactin levels were more likely for female elephants with higher Alternate Feeding Methods and Enrichment Diversity scores [12]. Finally, ideal body condition was associated with higher Walk Week scores and an unpredictable Feed Schedule [14]. The results of our study and the subsequent welfare analyses clearly illustrate that elephant management exists on a continuum, can be modified to support best practices, and are pertinent to elephant welfare.
